# Supplementary material for: Multiple conformations facilitate PilT function in the type IV pilus
Source: Nat Commun. 2019 Nov 15;10:5198. doi: 10.1038/s41467-019-13070-z (PMC6858323; doi:10.1038/s41467-019-13070-z)
Supplement: Supplementary file 1 — Supplementary Information [file 41467_2019_13070_MOESM1_ESM.pdf]

Supplementary information

Multiple conformations facilitate PilT function in the type IV pilus

Matthew McCallum *et al.*

Supplementary Table 1. Defining the open- and closed-interfaces across PilT/VirB11-like family member structures.

Protein (PDB)	PDB[Reference]	System	Inter-chain Cα distance (Å)* T221–H230	T221–L269	Corresponding Interface**	Pattern of Interfaces
Methylated PilT^{Gm}	6OJY[Herein]	T4aP	5.8<br>12.2	14.1<br>8.9	C<br>O	OCOCOC
Low ADP PilT^{Gm}	6OJZ[Herein]	T4aP	5.8<br>13.6	14.0<br>8.3	C<br>O	OCOCOC
High ADP PilT^{Gm}	6OK2[Herein]	T4aP	5.6<br>13.6	14.2<br>8.2	C<br>O	OCOCOC
ATP bound PilT^{Gm}	6OJX[Herein]	T4aP	6.1	12.3	C	CCCCCC
ANP/ADP bound PilT^{Gm}	6OKV[Herein]	T4aP	5.4<br>11.6	13.7<br>9	C<br>O	CCOCCO
PilT^{Pa}	3JVV[1]	T4aP	5.7	14.5	C	CCCCCC
PilT^{Pa}	3JVU[1]	T4aP	5.5	13.8	C	CCCCCC
PilT^{Aa}	2GSZ[2]	T4aP	7.6<br>15.8	19.4<br>9.9	C<br>O	OOCOOC
PilT^{Gs}	5ZFQ[3]	T4aP	12.0	6.5	O	OOOOOO
PilT^{Aa}	2EWV[2]	T4aP	11.2	13.7	X	XXXXXX
PilT^{Aa}	2EWW[2]	T4aP	10.9	13.7	X	XXXXXX
PilB^{Gm}	5TSH[4]	T4aP	5.9<br>22.5	20.6<br>10.0	C<br>O	CCOCCO
PilB^{Gm}	5TSG[4]	T4aP	6.6<br>21.3	20.2<br>9.3	C<br>O	CCOCCO
PilB^{Tt}	5IT5[5]	T4aP	6.1<br>20.6	20.0<br>9.8	C<br>O	CCOCCO
PilB^{Tt}	5OIU[6]	T4aP	6.1<br>21.4	20.2<br>9.8	C<br>O	CCOCCO
PilB^{Gs}	5ZFR[3]	T4aP	6.1<br>21.4	20.5<br>9	C<br>O	CCOCCO
GspE	4KSR[7]	T2S	7.4<br>21.4	19.6<br>9.4	C<br>O	CCOCCO
GspE	4KSS[7]	T2S	5.0	21.4	C	CCCCCC
FlaI	4II7[8]	Archaellum	8.6<br>14.7	19.5<br>7.9	C<br>O	OCOCOC
FlaI	4IHQ[8]	Archaellum	7.0<br>15.5	18.5<br>7.5	C<br>O	CCOCCO
Archaeal GspE	2OAP[9]	Archaeal T2S	7.8<br>11.4	16.1<br>8.8	C<br>O	OCOCOC
Archaeal GspE	2OAQ[9]	Archaeal T2S	7.6<br>11.9	16.1<br>9.8	C<br>O	OCOCOC
VirB11	1NLY[10]	T4aS	5.0	13.0	C	CCCCCC
VirB11	2PT7[11]	T4aS	6.0	14.3	C	CCCCCC
VirB11	1NLZ[10]	T4aS	5.0	13.0	C	CCCCCC
VirB11	1OPX[10]	T4aS	5.0	13.0	C	CCCCCC
DotB	6GEB[12]	T4bS	6.9<br>14.4	15.0<br>8.5	C<br>O	OCOCOC
DotB	6GEF[12]	T4bS	7<br>16.8	13.1<br>9.2	C<br>O	CCOCCO
**CryoEM structures presented herein**						
PilT^{Gm}	6OLL[Herein]	T4aP	5.4<br>11.4	13.6<br>7.7	C<br>O	OOCOOC
PilT^{Gm}	6OLK[Herein]	T4aP	6.0<br>12.6	13.4<br>7.9	C<br>O	OCOCOC
PilT^{Gm}	6OLM[Herein]	T4aP	6.2	12.4	C	CCCCCC
PilB^{Gm}	6OLJ[Herein]	T4aP	6.4<br>22.7	20.2<br>9.4	C<br>O	CCOCCO

*Mean distance reported in the case when multiple open- or closed-interfaces are present in the same hexamer. In homologs of PilT^{Gm}, residues that align with T221, H230, and L269 from PilT^{Gm} are used: respectively, T233, H242, and L281 for PDB 2EWV, 2EWW, and 2GSZ; T220, H229, and L268 for PDB 3JVV and 3JVU; T221, H230, and L269 for PDB 5ZFQ; T411, H420, and R455 for PDB 5TSH, 5TSG, 5ZFR, and 6OLJ; T735, H744, and R779 for PDB 5IT5; T245, H254, and R289 for PDB 5OIU; T251, H260, and L299 for PDB 6GEB; T266, H275, and L314 for PDB 6GEF; T350, H359, and R394 for PDB 4KSR and 4KSS; S263, H273, and H318 for PDB 1NLY, 2PT7, 1NLZ, and 1OPX; T356, H365, and M399 for PDB 2OAP and 2OAQ; and T351, H360, and L395 for PDB 4II7 and 4IHQ.

**The open-interface defined as T221–H230 distance > 12 Å and T221–L269 distance < 11 Å. The closed-interface defined as T221–H230 distance < 9 Å and T221–L269 distance > 11 Å.

Supplementary Table 2: Primers, strains, plasmids, and antibodies used in this study

**Primers**	**Sequence**	**Reference**
P168	TATATATACATATGGCCAACATGCATCAGC	This Study
P169	TATATATACTCGAGTTATCTCATGGGGGGGCG	This Study
P220	TATAATGGTACCTACCGAGCTGCTCGCCTT	This Study
P221	TATATATCTAGAATCAGAAGTTTTCCGGGATCTT	This Study
E204A_fwd	CATCCTGGTCGGCGCGATG	This Study
E204A_rev	CATCGCGCCGACCAGGATG	This Study
H229A_fwd	TCGGCACCCTGGCCACCACCTCG	This Study
H229A_rev	CGAGGTGGTGGCCAGGGTGCCGA	This Study
R239A_fwd	ACACGTCGACCACCGCGTCGATGGTCTTC	This Study
R239A_rev	GAAGACCATCGACGCGGTGGTCGACGTGT	This Study
E219K_fwd	GTGGCCGGTCTTCGCCGCGGTCA	This Study
E219K_rev	TGACCGCGGCGAAGACCGGCCAC	This Study
D31K_fwd	ATGATCCGGGTGAATGGCGATGTACG	This Study
D31K_rev	CGTACATCGCCATTCACCCGGATCAT	This Study
E258A_fwd	CTCGATGCTCTCCGCGTCGCTGCAAT	This Study
E258A_rev	ATTGCAGCGACGCGGAGAGCATCGAG	This Study
R294E_fwd	GCGCGACCTTGTCCTCCTCGATCAGGTTGCGGAT	This Study
R294E_rev	ATCCGCAACCTGATCGAGGAGGACAAGGTCGCGC	This Study
D242K_fwd	GGCCGGGAACACCTTGACCACCCGGTC	This Study
D242K_rev	GACCGGGTGGTCAAGGTGTTCCCGGCC	This Study
T216R_fwd	TCCGCCGCCCTCAGGGCCAGGCGGA	This Study
T216R_rev	TCCGCCTGGCCCTGAGGGCGGCGGA	This Study
R123D_fwd	ACCAGTACCAACCCGTCCGGGACGTCTGAAA	This Study
R123D_rev	TTTCAGACGTCCCGGACGGGTTGGTACTGGT	This Study
K58A_fwd	GACATCATGAACGACGCGCAGCGCAAGGACTTC	This Study
K58A_rev	GAAGTCCTTGCGCTGCGCGTCGTTCATGATGTC	This Study
K58Q_fwd	GACATCATGAACGACCAGCAGCGCAAGGACTTC	This Study
K58Q_rev	GAAGTCCTTGCGCTGCTGGTCGTTCATGATGTC	This Study
K58R_fwd	GACATCATGAACGACCGTCAGCGCAAGGACTTC	This Study
K58R_rev	GAAGTCCTTGCGCTGACGGTCGTTCATGATGTC	This Study
**Strains**	**Description**	**Reference**
E. coli TOP10	F^-, mcrA Δ(mrr-hsdRMS-mcrBC) ϕ80lacZΔM15 ΔlacX74 nupG recA1 araD139 Δ(ara-leu)7697 galE15 galK16 rpsL(Str^R) endA1 λ^-	Invitrogen
E. coli BL21-CodonPlus® cells	Expression strain; F^- *ompT hsdS* (rB^- mB^-) dcm^+ Tet^r gal λ (DE3) endA [*argU proL* Cam^r]	Stratagene
E. coli BTH101	BACTH coexpression strain; F^-, *cya-99, araD139, galE15, galK16, rpsL1, hsdR2, mcrA1, mcrB1*	Euromex
P. aeruginosa PAO1 *pilT*::FRT	Retraction deficient mutant with FRT disruption in *pilT* gene	[13]
**Plasmids**	**Description**	**Reference**
pET28a::$PilT^{PA}$	PilT from *P. aeruginosa* for expression in *E. coli*	[14]
pET28a::$PilB^{Gm}$	PilB from *G. metallireducens* for expression in *E. coli*	[4]
pET28a::$PilT^{Gm}$	PilT4 from *G. metallireducens* for expression in *E. coli*	This Study
pBADGr	pBAD plasmid with gentamicin resistance	[13]
pBADGr::$PilT^{PA}$<br>E204A, H229A, R239A, E219K, D31K, E258A, R294E, D242K, T216R, R123D, K58A, K58Q, K58R	PilT from *P. aeruginosa* for expression in *P. aeruginosa*<br>pBADGr::$PilT^{PA}$ constructs with individual site mutations	This Study<br>This Study
pUT18C:$PilC^{PA}$	PilC from *P. aeruginosa* BACTH construct	[15]
pUT18C:$PilT^{PA}$	PilT from *P. aeruginosa* BACTH construct	[15]
pKT25:$PilT^{PA}$<br>E204A, H229A, R239A, E219K, D31K, E258A, R294E, D242K, T216R, R123D, K58A, K58Q, K58R	PilT from *P. aeruginosa* BACTH construct<br>pKT25:$PilT^{PA}$ constructs with individual site mutations	[15]<br>This Study
**Antibodies**	**Description**	**Reference**
α-PilT	Purified rabbit IgG antibody made with PilT from *P. aeruginosa*	[16]

Supplementary Table 3. Data collection and refinement statistics of PilT crystal structures

**Crystal structure name**	**Methylated OCOCOC PilT**	**Partial occupancy ADP OCOCOC PilT**	**Full occupancy ADP OCOCOC PilT**	**CCCCCC PilT**	**CCOCCO PilT**
Relevant ligand	SO_4^{2-}	Partial occupancy ADP	Full occupancy ADP	ATP	ANP and ADP
**Data collection**					
Facility	CLS	NSLS-II	NSLS-II	APS	CLS
Beamline	08-ID-1	17-ID-2	17-ID-1	23-ID-B	08-ID-1
Wavelength (Å)	0.97949	1.282140	0.999614	1.033202	0.97949
Space group	$P2_12_12_1$	$P2_12_12_1$	$P2_12_12_1$	$P6$	$P2_12_12_1$
a, b, c (Å)	115.8, 119.01, 178.3	112.3, 121.1, 187.1	111.8 121.0 185.9	190.2, 190.2, 60.5	98.5, 127.1, 187.3
α, β, γ (°)	90, 90, 90	90, 90, 90	90, 90, 90	90, 90, 120	90, 90, 90
Resolution (Å)	48-3.3 (3.4-3.3)	29-3.0 (3.1-3.0)	29-3.3 (3.4-3.3)	48-1.9 (2.0-1.9)	48-4.0 (4.1-4.0)
Total Reflections	281393	620489	258149	1037608	127072
Unique Reflections	37753 (3658)	49747 (4342)	38771 (3597)	99891(9942)	38507 (1745)
Redundancy	7.4 (7.6)	12.5 (12.1)	6.7 (6.0)	10.4 (10.3)	3.3 (0.6)
Completeness (%)	100 (99)	98 (85)	99 (94)	100 (100)	98 (10)*
Mean $I / \sigma I$	12.6 (2.3)	12 (2.2)	7.5 (1.3)	10.6 (1.3)	4.0 (1.6)
R_{Sym} (%)**	16 (105)	16 (84)	16 (110)	12 (143)	28 (112)
CC* (%)**	100 (93)	100 (95)	100 (87)	100 (89)	100 (86)
**Refinement**					
R_{work} / R_{free} (%)†	21.0 / 24.5	19.3 / 23.1	21.2 / 25.43	18.4 / 21.2	24.8 / 27.6
RMSD					
Bond lengths (Å)	0.005	0.009	0.008	0.011	0.009
Bond angles (°)	0.90	1.03	0.84	0.88	1.12
Ramachandran‡					
Favoured (%)	98	98	98	98	98
Allowed (%)	100	100	100	100	100
Coordinate error (Å)§	0.34	0.35	0.52	0.21	0.48
Atoms	16457	16569	16205	9562	16204
Protein	16236	16357	16031	8335	16010
Water	191	50	12	1099	12
Magnesium	0	0	0	3	4
ANP	0	0	0	0	124
ATP	0	0	0	93	0
ADP	0	162	162	0	54
Sulfate	30	0	0	0	0
Ethylene Glycol	0	0	0	32	0
Av. B-factors (Å^2)	72.6	81.3	97.6	44.2	86.4
Protein	73.2	81.3	97.5	43.1	86.4
Water	26.1	65.7	70.8	54.7	84.6
Ligands	45.8	89.9	119	35.7	87.1
**Deposited density and coordinate files**					
PDB ID	6OJY	6OJZ	6OK2	6OJX	6OKV

Note: Values in parentheses correspond to the highest resolution shell.

*, Atypical completeness of the CCOCCO PilT structures reflects anisotropic truncation

**, $R_{Sym} = \Sigma \Sigma \mid I - (I) \mid / \Sigma \Sigma I$, $R_{Pim} = \Sigma \sqrt{}(1/(n-1) \Sigma \mid I - (I) \mid / \Sigma \Sigma I$, and $CC^* = \sqrt{}\ (2CC_{1/2}/(1+CC_{1/2}))$ where $CC_{1/2}$ is the Pearson correlation coefficient of two half data sets as described elsewhere[17]

†, $R_{work} = \Sigma \mid |F_{obs}| - k|F_{calc}| \mid / |F_{obs}|$ where F_{obs} and F_{calc} are the observed and calculated structure factors, respectively. R_{free} is the sum extended over a subset of reflections (5%) excluded from all stages of the refinement

§, Maximum-likelihood based Coordinate Error, as determined by *PHENIX*[18]

Supplementary Table 4. CryoEM data acquisition, processing, atomic model statistics and map/model depositions.

**Data collection**	**Tilted data collection**	**All other samples**
Electron Microscope	Titan Krios	FEI Tecnai F20
Tilt angle	40°	0°
Camera	Falcon 3EC	Gatan K2 Summit
Voltage (kV)	300	200
Nominal Magnification	75000	25000
Calibrated Pixel size (Å)	1.06	1.45
Exposure rate (e/pixel/s)	0.8	5
Exposure (e/Å^2)	42.7	35
Frames	30	30
**Image processing**	**Tilted data collection**	**All other samples**
Motion correction software	cryoSPARC v2	cryoSPARC v2
CTF estimation software	CTFFIND4 (refined with GCTF)	CTFFIND4
Particle selection software	cryoSPARC v2	cryoSPARC v2
Classification and refinement software	cryoSPARC v2	cryoSPARC v2

**Sample**	**PilT^{Gm}** Tilted data	**PilT^{Gm}** No nucleo-tide added	**PilT^{Gm}** 1 mM ATP	**PilT^{Gm}** 0.1 mM ADP	**PilT^{Gm}** 1 mM ADP	**PilT^{Gm}** 1 mM ATP & 0.1 mM ADP	**PilT^{Gm}** 1 mM ATP & 1 mM ADP	**PilT^{Pa}** No nucleo-tide added	**PilT^{Pa} K58A** No nucleo-tide added	**PilB^{Gm}** No nucleo-tide added
Micrographs	570	64	46	21	31	7	14	18	43	15
Selected particles	286433	35025	52331	3668	36814	3438	10990	22230	54715	7394

**EM maps**	**PilT^{Gm} Tilted data** OOCOOC	OCOCOC	**PilT^{Gm} + 1 mM ATP** CCCCCC	**PilB^{Gm}** CCOCCO
Particle images contributing to maps	100786	94151	43823	3257
Applied symmetry	C_2	C_3	C_6	C_2
Applied B-factor (Å^2)	177	179	95	498
Global resolution (FSC = 0.143, Å)	4.1	4.0	4.4	7.8
**Model building**	**PilT^{Gm} Tilted data** OOCOOC	OCOCOC	**PilT^{Gm} + 1 mM ATP** CCCCCC	**PilB^{Gm}** CCOCCO
Modeling software	Coot, Phenix	Coot, Phenix	Coot, Phenix	Coot, Phenix
Atoms	10426	10323	10416	11442
Protein	10426	10323	10416	11442
ATP	0	0	186	0
RMSD<br>Bond length (Å)<br>Bond angle (°)	 0.007<br>1.06	 0.005<br>1.05	 0.006<br>1.05	 0.007<br>1.43
Ramachandaran<br>Favoured (%)<br>Allowed	 98<br>99.8	 98<br>100	 98<br>100	 98<br>100
Clashscore	0.4	0.2	0.92	2.46
Av. B-factor (Å^2)<br>Protein<br>Ligand	 6.4<br>-	 3.1<br>-	 22.2<br>24.6	 68.8<br>-
MolProbity score‡	0.70	0.65	0.78	1.03
CC_{mask}	0.50	0.46	0.57	0.42
CC_{Ligand}	-	-	0.67	-
**Deposited maps and coordinate files**				
EMDB code	EMD-20116	EMD-20115	EMD-20117	EMD-20114
PDB code	6OLL	6OLK	6OLM	6OLJ

‡, As calculated using *MolProbity*[19] implemented in *PHENIX*

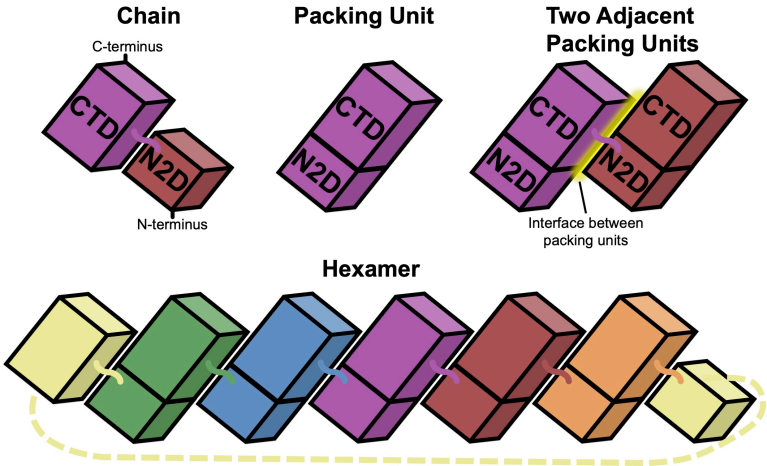

Supplementary Figure 1 – Nomenclature used herein. Each PilT chain is composed of two domains: N2D and CTD. The packing unit is composed of the $N2D^{n}$ and CTD^{n+1}. Two adjacent packing units contact one-another creating an interface between packing units. Six packing units organize in this manner to create a hexamer.

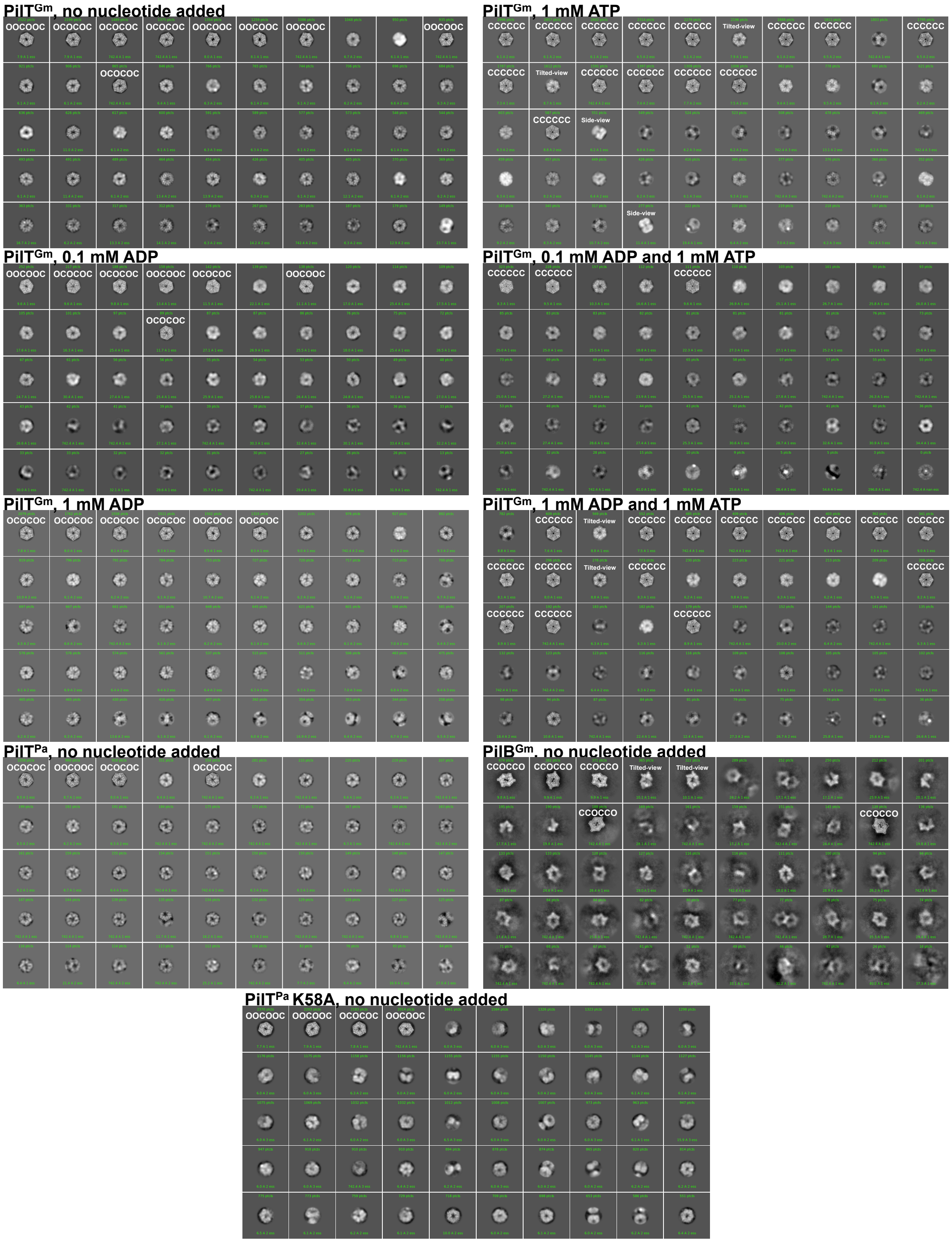

Supplementary Figure 2 – 2D class averages of PilB and PilT particles and their proposed conformation. The protein and nucleotide added are indicated above each list of 50 class averages. Class averages are sorted by the number of particles in each class. The qualitative assignment of the class average conformation is noted above each class average in white. A dotted outline of the conformation cartoon from Figure 4a is overlaid on the 2D class average for reference. Source data (without dotted outlines) are provided as a Source Data file. Class averages that were too blurry to identify the conformation are not annotated. Class averages that appear to represent tilted-views or side views are also labeled in white.

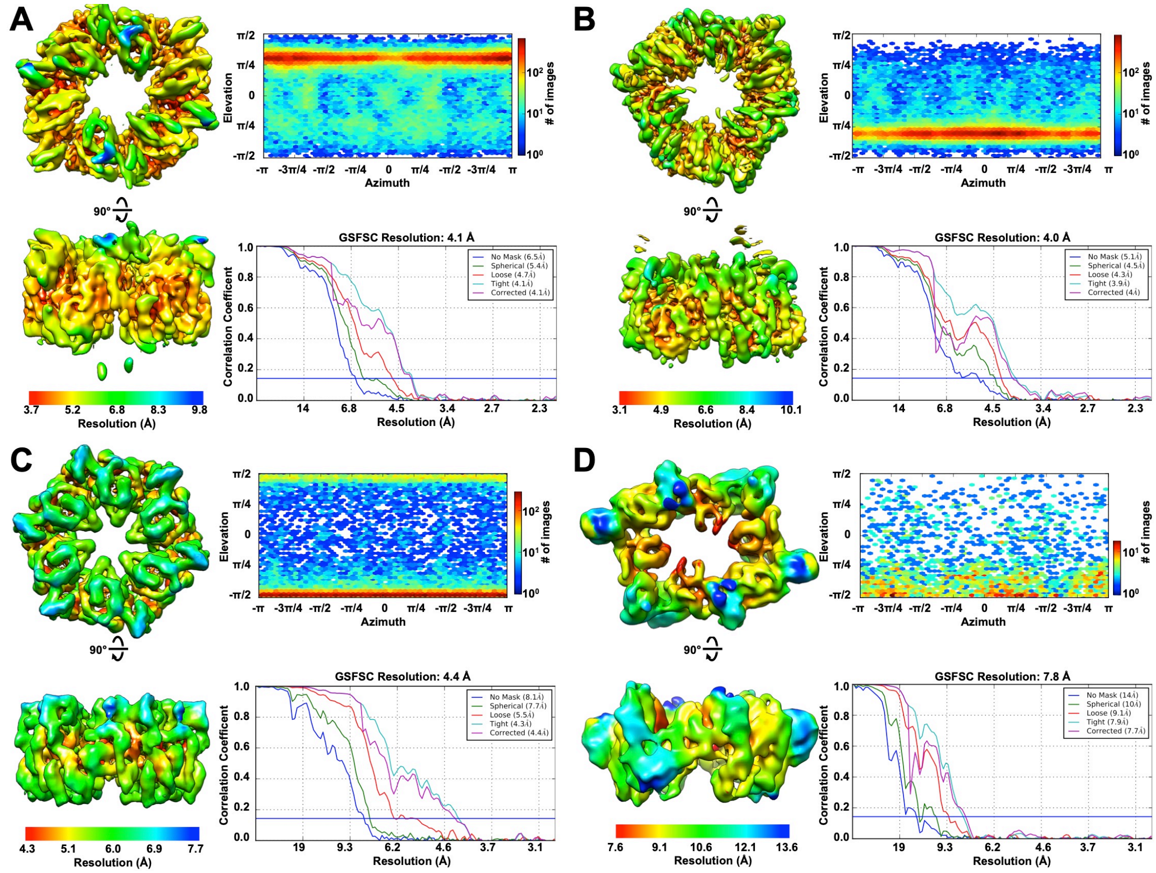

Supplementary Figure 3 – Local resolution (left), particle distribution (upper right), and Fourier shell correlation (FSC) curves (lower right). **a**, Locally sharpened PilT^{Gm} OOCOOC conformation map. **b**, Locally sharpened PilT^{Gm} OCOCOC conformation map. **c**, Locally sharpened PilT^{Gm} CCCCCC conformation map. **d**, Locally sharpened PilB^{Gm} CCOCCO conformation map.

SUPPLEMENTARY REFERENCES

1. Misic, A.M., Satyshur, K.A. & Forest, K.T. P. aeruginosa PilT structures with and without nucleotide reveal a dynamic type IV pilus retraction motor. *J Mol Biol* **400**, 1011-21 (2010).
2. Satyshur, K.A. et al. Crystal structures of the pilus retraction motor PilT suggest large domain movements and subunit cooperation drive motility. *Structure* **15**, 363-76 (2007).
3. Solanki, V., Kapoor, S. & Thakur, K.G. Structural insights into the mechanism of Type IVa pilus extension and retraction ATPase motors. *FEBS J* **285**, 3402-3421 (2018).
4. McCallum, M., Tammam, S., Khan, A., Burrows, L.L. & Howell, P.L. The molecular mechanism of the type IVa pilus motors. *Nat Commun* **8**, 15091 (2017).
5. Mancl, J.M., Black, W.P., Robinson, H., Yang, Z. & Schubot, F.D. Crystal Structure of a Type IV Pilus Assembly ATPase: Insights into the Molecular Mechanism of PilB from Thermus thermophilus. *Structure* **24**, 1886-1897 (2016).
6. Collins, R. et al. Structural cycle of the Thermus thermophilus PilF ATPase: the powering of type IVa pilus assembly. *Sci Rep* **8**, 14022 (2018).
7. Lu, C. et al. Hexamers of the type II secretion ATPase GspE from Vibrio cholerae with increased ATPase activity. *Structure* **21**, 1707-17 (2013).
8. Reindl, S. et al. Insights into FlaI functions in archaeal motor assembly and motility from structures, conformations, and genetics. *Mol Cell* **49**, 1069-82 (2013).
9. Yamagata, A. & Tainer, J.A. Hexameric structures of the archaeal secretion ATPase GspE and implications for a universal secretion mechanism. *EMBO J* **26**, 878-90 (2007).
10. Savvides, S.N. et al. VirB11 ATPases are dynamic hexameric assemblies: new insights into bacterial type IV secretion. *EMBO J* **22**, 1969-80 (2003).
11. Hare, S. et al. Identification, structure and mode of action of a new regulator of the Helicobacter pylori HP0525 ATPase. *EMBO J* **26**, 4926-34 (2007).
12. Prevost, M.S. & Waksman, G. X-ray Crystal Structures of the Type IVb Secretion System DotB ATPases. *Protein Sci* **27**, 1464-75 (2018).
13. Asikyan, M.L., Kus, J.V. & Burrows, L.L. Novel proteins that modulate type IV pilus retraction dynamics in Pseudomonas aeruginosa. *J Bacteriol* **190**, 7022-34 (2008).
14. Chiang, P. et al. Functional role of conserved residues in the characteristic secretion NTPase motifs of the Pseudomonas aeruginosa type IV pilus motor proteins PilB, PilT and PilU. *Microbiology* **154**, 114-26 (2008).
15. McCallum, M. et al. PilN Binding Modulates the Structure and Binding Partners of the Pseudomonas aeruginosa Type IVa Pilus Protein PilM. *J Biol Chem* **291**, 11003-15 (2016).
16. Takhar, H.K., Kemp, K., Kim, M., Howell, P.L. & Burrows, L.L. The platform protein is essential for type IV pilus biogenesis. *J Biol Chem* **288**, 9721-8 (2013).
17. Karplus, P.A. & Diederichs, K. Linking crystallographic model and data quality. *Science* **336**, 1030-3 (2012).
18. Adams, P.D. et al. PHENIX: a comprehensive Python-based system for macromolecular structure solution. *Acta Crystallogr D Biol Crystallogr* **66**, 213-21 (2010).
19. Chen, V.B. et al. MolProbity: all-atom structure validation for macromolecular crystallography. *Acta Crystallogr D Biol Crystallogr* **66**, 12-21 (2010).
