# Supplementary material for: Multiple conformations facilitate PilT function in the type IV pilus
Source: Nat Commun. 2019 Nov 15;10:5198. doi: 10.1038/s41467-019-13070-z (PMC6858323; doi:10.1038/s41467-019-13070-z)
Supplement: Supplementary file 3 — Reporting Summary [file 41467_2019_13070_MOESM3_ESM.pdf]

nature research

Corresponding author(s): John L. Rubinstein, Lori L. Burrows, and P. Lynne Howell

Last updated by author(s): Jul 16, 2019

Reporting Summary

Nature Research wishes to improve the reproducibility of the work that we publish. This form provides structure for consistency and transparency in reporting. For further information on Nature Research policies, see Authors & Referees and the Editorial Policy Checklist .

Statistics

For all statistical analyses, confirm that the following items are present in the figure legend, table legend, main text, or Methods section.

n/a	Confirmed	
☐	☒	The exact sample size (*n*) for each experimental group/condition, given as a discrete number and unit of measurement
☐	☒	A statement on whether measurements were taken from distinct samples or whether the same sample was measured repeatedly
☐	☒	The statistical test(s) used AND whether they are one- or two-sided<br>*Only common tests should be described solely by name; describe more complex techniques in the Methods section.*
☐	☒	A description of all covariates tested
☐	☒	A description of any assumptions or corrections, such as tests of normality and adjustment for multiple comparisons
☐	☒	A full description of the statistical parameters including central tendency (e.g. means) or other basic estimates (e.g. regression coefficient) AND variation (e.g. standard deviation) or associated estimates of uncertainty (e.g. confidence intervals)
☐	☒	For null hypothesis testing, the test statistic (e.g. *F*, *t*, *r*) with confidence intervals, effect sizes, degrees of freedom and *P* value noted<br>*Give P values as exact values whenever suitable.*
☒	☐	For Bayesian analysis, information on the choice of priors and Markov chain Monte Carlo settings
☒	☐	For hierarchical and complex designs, identification of the appropriate level for tests and full reporting of outcomes
☒	☐	Estimates of effect sizes (e.g. Cohen's *d*, Pearson's *r*), indicating how they were calculated

Our web collection on statistics for biologists contains articles on many of the points above.

Software and code

Policy information about availability of computer code

Data collection	MxLive and LSDC (for crystal diffraction collection); EPU (for cryoEM data collection)
Data analysis	XDS, Phenix, and Coot (for crystal data processing, model building, and refinement); cryosparc v2, Chimera, Phenix, and Coot (for cryoEM data processing, model building, and refinement); Pymol and Chimera (for 3D model visualization); CMview (to identify residues that contact one another); EVcouplings Webserver (for identifying evolutionarily coupled residues)

For manuscripts utilizing custom algorithms or software that are central to the research but not yet described in published literature, software must be made available to editors/reviewers. We strongly encourage code deposition in a community repository (e.g. GitHub). See the Nature Research guidelines for submitting code & software for further information.

Data

Policy information about availability of data

All manuscripts must include a data availability statement. This statement should provide the following information, where applicable:

- Accession codes, unique identifiers, or web links for publicly available datasets
- A list of figures that have associated raw data
- A description of any restrictions on data availability

PDB (http://www.rcsb.org/) and EMDB (https://www.ebi.ac.uk/pdbe/emdb/)
Figures 1-3 --- PDB ID: 6OJY, 6OJZ, 6OK2, 6OJX, 6OKV
Figures 3-4 --- PDB ID (EMDB): 6OLL (EMD-20116), 6OLK (EMD-20115), 6OLM (EMD-20117), 6OLJ (EMD-20114)
No restrictions

Field-specific reporting

Please select the one below that is the best fit for your research. If you are not sure, read the appropriate sections before making your selection.

☒ Life sciences ☐ Behavioural & social sciences ☐ Ecological, evolutionary & environmental sciences

For a reference copy of the document with all sections, see nature.com/documents/nr-reporting-summary-flat.pdf

Life sciences study design

All studies must disclose on these points even when the disclosure is negative.

Sample size	Twitching assays were done in 6 replicates (independent colonies used to inoculate distinct plates); triplicate is standard here, but six replicates were chosen to be more certain of the observed phenotypes. The BACTH assays were done in triplicate as is standard.
Data exclusions	N/A
Replication	All attempts at replicating the experimental findings were successful
Randomization	Not relevant; study used purified proteins and homogeneous populations of bacteria
Blinding	Blinding is not typical for structural and biochemical experiments

Reporting for specific materials, systems and methods

We require information from authors about some types of materials, experimental systems and methods used in many studies. Here, indicate whether each material, system or method listed is relevant to your study. If you are not sure if a list item applies to your research, read the appropriate section before selecting a response.

Materials & experimental systems

n/a	Involved in the study	
☐	☒	Antibodies
☒	☐	Eukaryotic cell lines
☒	☐	Palaeontology
☒	☐	Animals and other organisms
☒	☐	Human research participants
☒	☐	Clinical data

Methods

n/a	Involved in the study	
☒	☐	ChIP-seq
☒	☐	Flow cytometry
☒	☐	MRI-based neuroimaging

Antibodies

Antibodies used	anti-PilT
Validation	Purified rabbit IgG antibody made with PilT from P. aeruginosa; Takhar, H.K., Kemp, K., Kim, M., Howell, P.L. & Burrows, L.L. The platform protein is essential for type IV pilus biogenesis. J Biol Chem 288, 9721-8 (2013).
